# Supplementary material for: Exploring the Papillomaviral Proteome to Identify Potential Candidates for a Chimeric Vaccine against Cervix Papilloma Using Immunomics and Computational Structural Vaccinology
Source: Viruses. 2019 Jan 15;11(1):63. doi: 10.3390/v11010063 (PMC6357041; doi:10.3390/v11010063)
Supplement: Supplementary file 1 [file viruses-11-00063-s001.zip › supplementary material/Viruses-407150_SFigures_Revised.pdf]

Exploring papillomaviral proteome to identify potential candidates for chimeric vaccine against cervix papilloma using immunomics and computational structural vaccinology

Satyavani Kaliamurthi[1,2], Gurudeeban Selvaraj[1,2], Sathishkumar Chinnasamy[3], Qiankun Wang[3], Asma Sindhoo Nangraj[3], William C Cho[4], Keren Gu[1,2], Dong-Qing Wei[1,3*]

[1]Center of Interdisciplinary Science-Computational Life Sciences, College of Food Science and Engineering, Henan University of Technology, Zhengzhou High-tech Industrial Development Zone, 100 Lianhua Street, Zhengzhou, Henan 450001, China

[2]College of Chemistry, Chemical Engineering and Environment, Henan University of Technology, Zhengzhou High-tech Industrial Development Zone, 100 Lianhua Street, Zhengzhou, Henan 450001, China.

[3]The State Key Laboratory of Microbial Metabolism, College of Life Sciences and Biotechnology, Shanghai Jiao Tong University, No: 800 Dongchuan Road, Minhang, Shanghai, 200240, China

[4]Department of Clinical Oncology, Queen Elizabeth Hospital, Kowloon, Hong Kong

Corresponding Author: Prof. Dong-Qing Wei, Department of Bioinformatics, The State Key Laboratory of Microbial Metabolism, College of Life Sciences and Biotechnology, Shanghai Jiao Tong University, No: 800 Dongchuan Road, Minhang, Shanghai, 200240, China, Email: dqwei@sjtu.edu.cn; Tel.: +86 021 3420 4717; Fax: +86 021 3420 5709

Supplementary Figures

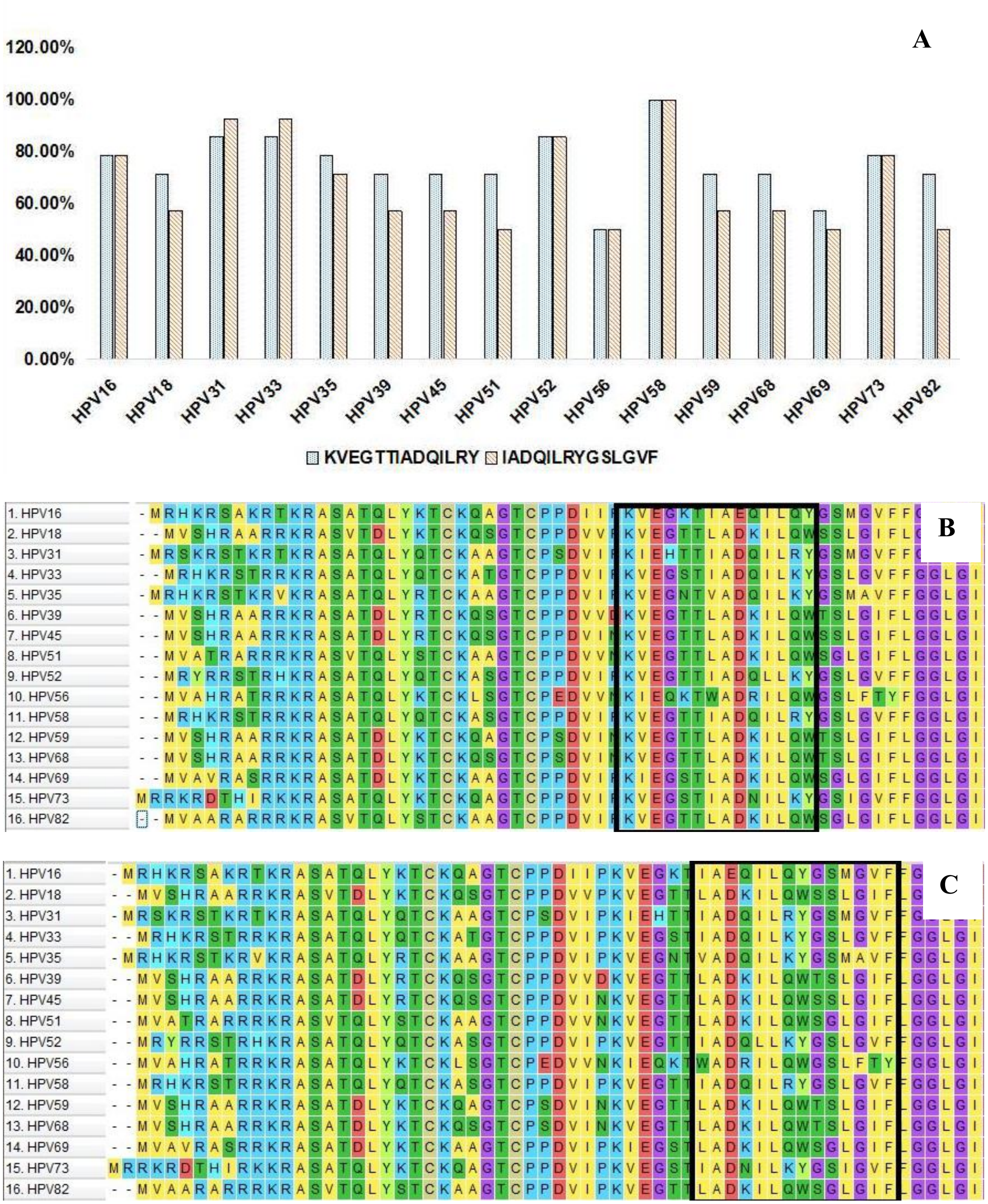

Figure S1 Conservation of two overlapped epitope segments in fifteen hrHPV strains. (a) Conservancy analysis by using Epitope conservancy analysis tool. Fifteen hrHPV strains conserved epitope segments were represented in X-axis; Percentage (%) of epitope conservancy among the hrHPV strains were showed in Y-axis. The sequence conservation of overlapped epitope segments of HPV58 as (b) KVEGTTIADQILRY

23-36 and (c) IADQILRYGSLGVF 29-42 was done using MEGA v7.0. Clustal analysis was analyzed using USCF Chimera. The epitope segments are represented by black rectangular boxes.

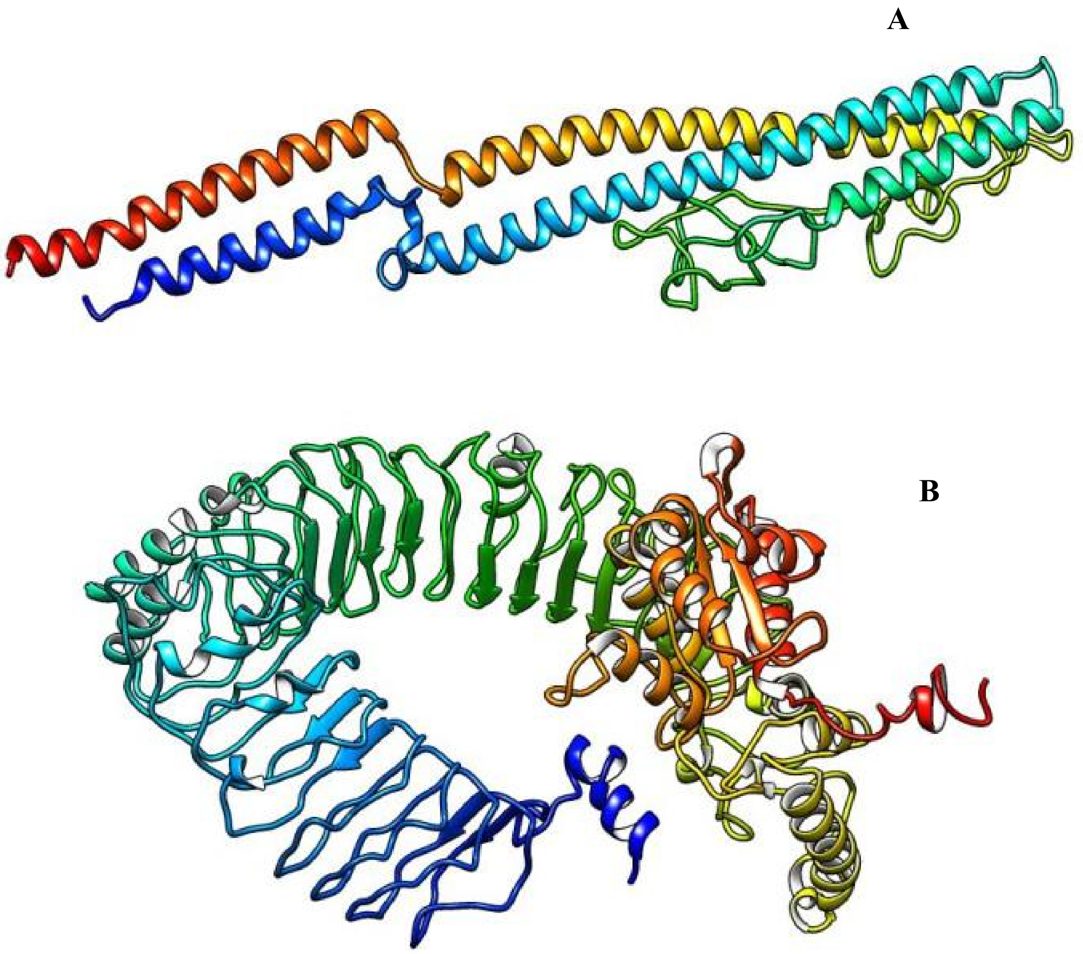

Figure S2 Refined 3D structure of the VC58 and TLR5 by using UCSF Chimera (a) The 3D structure of the VC58 was obtained through homology modeling by using Robetta (b) The 3D structure of the mouse TLR5 was obtained through homology modeling by using Robetta

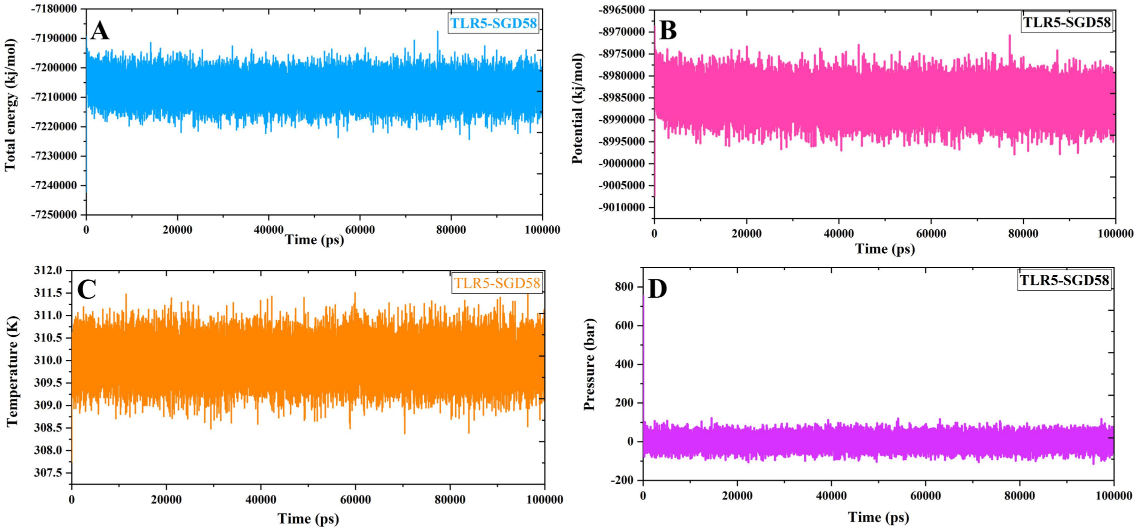

Figure S3 (a) Total energy, (b) potential energy, (c) temperature and (d) pressure plots of MD simulation for TLR5- VC58 complex in simulations of 100 ns.

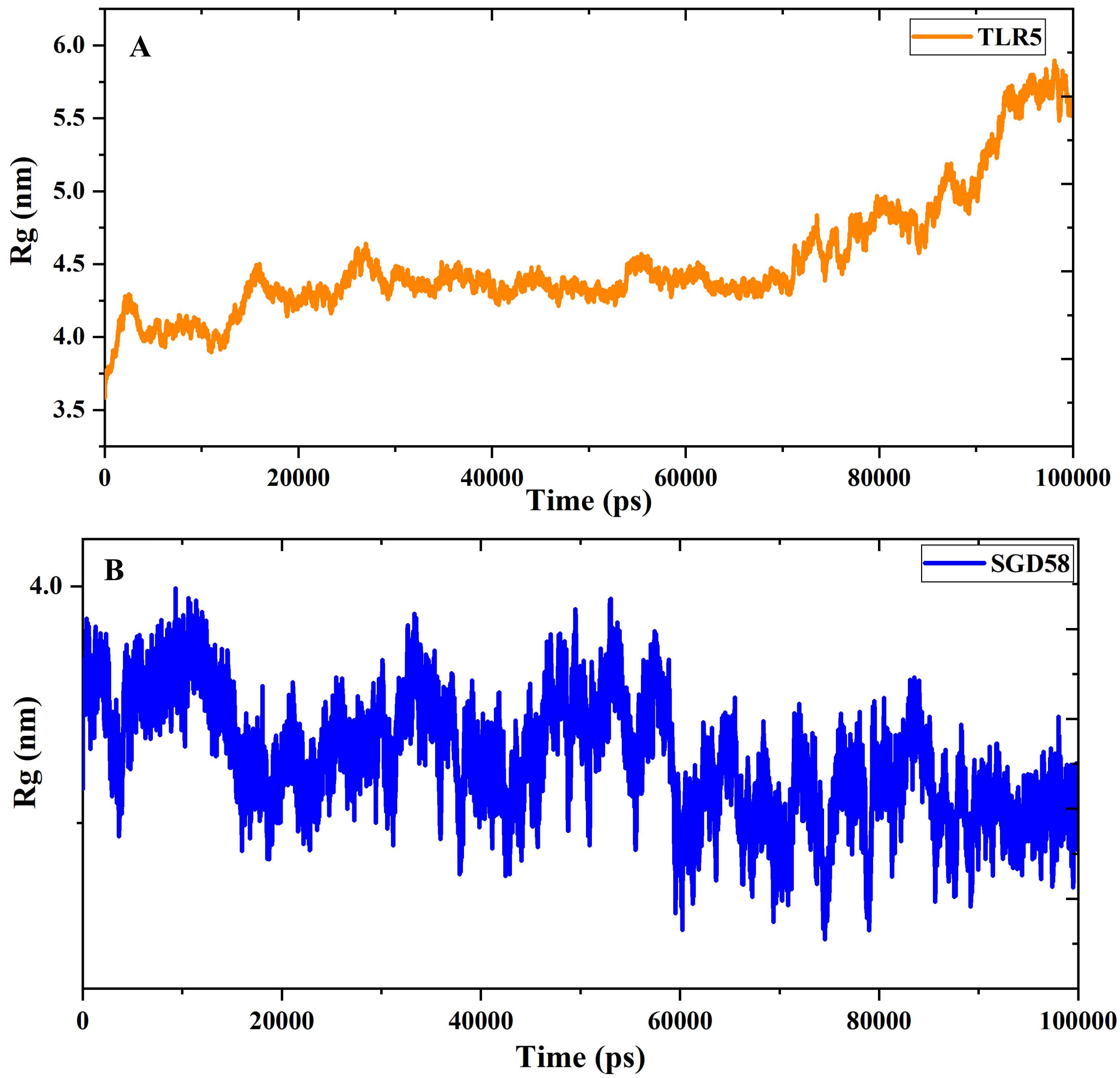

Figure S4 (a) Rg plot of vaccine molecule and (b) Rg plot of complex vaccine molecule.

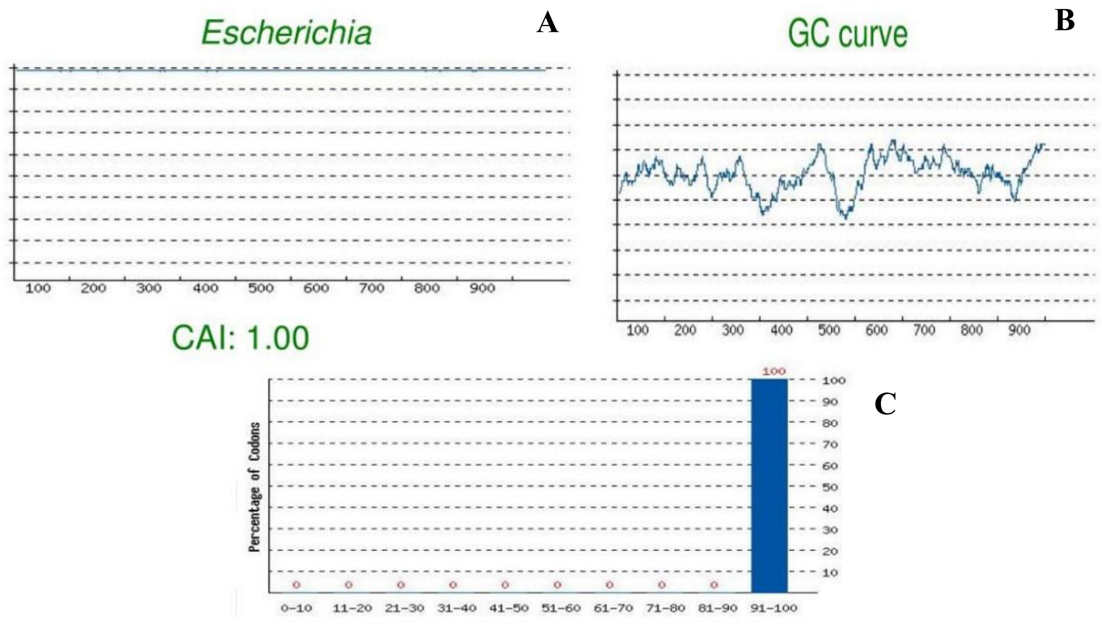

Figure S5 Codon optimization and *in silico* cloning of the gene. (a) The gene (reverse translated coding sequence of the vaccine construct) having ideal CAI value of 1.00 (>0.8), which is more suitable for higher expression in the *E. coli* host organism (b) The percentage of GC content in the gene is 59.49% , which is in the ideal range of GC content (between 30 to 70%) (c) CFD value of the gene is 100%. The value of 100 is set for the codon with the highest usage frequency for a given amino acid in the desired expression organism.
