# Supplementary material for: Exploring the Papillomaviral Proteome to Identify Potential Candidates for a Chimeric Vaccine against Cervix Papilloma Using Immunomics and Computational Structural Vaccinology
Source: Viruses. 2019 Jan 15;11(1):63. doi: 10.3390/v11010063 (PMC6357041; doi:10.3390/v11010063)
Supplement: Supplementary file 1 [file viruses-11-00063-s001.zip › supplementary material/Viruses-407150_STables_Revised.pdf]

Exploring papillomaviral proteome to identify potential candidates for chimeric vaccine against cervix papilloma using immunomics and computational structural vaccinology

Satyavani Kaliamurthi[1,2], Gurudeeban Selvaraj[1,2], Sathishkumar Chinnasamy[3], Qiankun Wang[3], Asma Sindhoo Nangraj[3], William C Cho[4],

Keren Gu[1,2], Dong-Qing Wei[1,3*]

[1]Center of Interdisciplinary Science-Computational Life Sciences, College of Food Science and Engineering, Henan University of Technology, Zhengzhou High-tech Industrial Development Zone, 100 Lianhua Street, Zhengzhou, Henan 450001, China

[2]College of Chemistry, Chemical Engineering and Environment, Henan University of Technology, Zhengzhou High-tech Industrial Development Zone, 100 Lianhua Street, Zhengzhou, Henan 450001, China.

[3]The State Key Laboratory of Microbial Metabolism, College of Life Sciences and Biotechnology, Shanghai Jiao Tong University, No: 800 Dongchuan Road, Minhang, Shanghai, 200240, China

[4]Department of Clinical Oncology, Queen Elizabeth Hospital, Kowloon, Hong Kong

Supplementary Tables

Table S1 . The overlapped epitope segments of MHC-I, CTL and TCR from N-terminal region of HPV58 were predicted by using different servers

MHC-1		CTL	TCR-peptide/peptide-MHC interfaces
IEDB[a]	NetMHC 4.0[b]	CTLPred[c]	PAComplex[d]
23-36	23-35	16-24	19-27
30-43	29-42	40-48	38-46
10-23	9-22	16-24<br>4-12	4-12
29-42	28-41	38-46	38-46

MHC-I overlapped epitope segments prediction by using different tools as [a]IEDB consensus and [b]NetMHCV4.0; CTL epitopes prediction by using CTLPred; TCR-peptide and peptide-MHC interface predicted by PAComplex

Table S2. The overlapped epitope segments of MHC-II, INF-gamma producing and B-cell epitopes N-terminal region of HPV58 by using different servers

MHC-II		INF-ɤ producing epitopes	B-cell epitopes
[a]IEDB consensus	[b]Tepitool	[c]INFepitope	[d]ABCPred
23-36	23-36	0.51	26-41
23-37	23-37	0.53	
29-43	29-43	+1	26-41
30-44	30-44	+1	
7-21	7-21	+1	7-22
6-20	6-20	+1	
29-43	29-43	+1	33-48
28-42	28-42	+1	

MHC-II overlapped epitope segments prediction by using different tools as [a]IEDB consensus and [b]Tepitool; INF-ɤ production of the overlapped epitope segments by using INFepitope; Overlapped B-cell linear epitope segments prediction by using ABCPred

Table S3. Conservation across-hrHPV strains by the overlapped HPV58 epitope segments

S.No.	Epitopes	Positions	Protein sub sequences	Identity (%)	Name of the Strain
1	CKASGTCPPDVIPK	21-34	CKASGTCPPDVIPK	100.00%	HPV52
2		21-34	CKASGTCPPDVIPK	100.00%	HPV58
3		21-34	CKATGTCPPDVIPK	92.86%	HPV33
4		22-35	CKAAGTCPPDVIPK	92.86%	HPV35
5		21-34	CKAAGTCPPDVIPK	92.86%	HPV69
6		21-34	CKAAGTCPPDVIPK	92.86%	HPV82
7		21-34	CKQSGTCPPDVVPK	85.71%	HPV18
8		22-35	CKAAGTCPSDVIPK	85.71%	HPV31
9		21-34	CKQSGTCPPDVINK	85.71%	HPV45
10		23-36	CKQAGTCPPDVIPK	85.71%	HPV73
11		22-35	CKQAGTCPPDIIPK	78.57%	HPV16
12		21-34	CKQSGTCPPDVVDK	78.57%	HPV39
13		21-34	CKAAGTCPPDVVNK	78.57%	HPV51
14		21-34	CKQSGTCPSDVINK	78.57%	HPV68
15		21-34	CKLSGTCPEDVVNK	71.43%	HPV56
16		21-34	CKQAGTCPSDVINK	71.43%	HPV59
1	KVEGTTIADQILRY	34-47	KVEGTTIADQILRY	100.00%	HPV58
2		35-48	KIEHTTIADQILRY	85.71%	HPV31
3		34-47	KVEGSTIADQILKY	85.71%	HPV33
4		34-47	KVEGTTIADQLLKY	85.71%	HPV52
5		35-48	KVEGKTIAEQILQY	78.57%	HPV16
6		35-48	KVEGNTVADQILKY	78.57%	HPV35
7		36-49	KVEGSTIADNILKY	78.57%	HPV73
8		34-47	KVEGTTLADKILQW	71.43%	HPV18
9		34-47	KVEGTTLADKILQW	71.43%	HPV39
10		34-47	KVEGTTLADKILQW	71.43%	HPV45
11		34-47	KVEGTTLADKILQW	71.43%	HPV51

12		34-47	KVEGTTLADKILQW	71.43%	HPV59
13		34-47	KVEGTTLADKILQW	71.43%	HPV68
14		34-47	KVEGTTLADKILQW	71.43%	HPV82
15		34-47	KIEGSTLADKILQW	57.14%	HPV69
16		34-47	KIEQKTWADRILQW	50.00%	HPV56
	**IADQILRYGSLGVF**	40-53	IADQILRYGSLGVF	100.00%	HPV58
1		41-54	IADQILRYGSMGVF	92.86%	HPV31
2		40-53	IADQILKYGSLGVF	92.86%	HPV33
3		40-53	IADQLLKYGSLGVF	85.71%	HPV52
4		41-54	IAEQILQYGSMGVF	78.57%	HPV16
5		42-55	IADNILKYGSIGVF	78.57%	HPV73
6		41-54	VADQILKYGSMAVF	71.43%	HPV35
7		40-53	LADKILQWSSLGIF	57.14%	HPV18
8		40-53	LADKILQWTSLGIF	57.14%	HPV39
9		40-53	LADKILQWSSLGIF	57.14%	HPV45
10		40-53	LADKILQWTSLGIF	57.14%	HPV59
11		40-53	LADKILQWTSLGIF	57.14%	HPV68
12		40-53	LADKILQWSGLGIF	50.00%	HPV51
13		40-53	WADRILQWGSLFTY	50.00%	HPV56
14		40-53	LADKILQWSGLGIF	50.00%	HPV69
15		40-53	LADKILQWSGLGIF	50.00%	HPV82
16		40-53	IADQILRYGSLGVF	100.00%	HPV58
1	**ADQILRYGSLGVFF**	41-54	ADQILRYGSLGVFF	100.00%	HPV58
2		42-55	ADQILRYGSMGVFF	92.86%	HPV31
3		41-54	ADQILKYGSLGVFF	92.86%	HPV33
4		41-54	ADQLLKYGSLGVFF	85.71%	HPV52
5		42-55	AEQILQYGSMGVFF	78.57%	HPV16
6		42-55	ADQILKYGSMAVFF	78.57%	HPV35
7		43-56	ADNILKYGSIGVFF	78.57%	HPV73
8		41-54	ADKILQWSSLGIFL	57.14%	HPV18
9		41-54	ADKILQWTSLGIFL	57.14%	HPV39
10		41-54	ADKILQWSSLGIFL	57.14%	HPV45

11	41-54	ADRILQWGSLFTYF	57.14%	HPV56
12	41-54	ADKILQWTSLGIFL	57.14%	HPV59
13	41-54	ADKILQWTSLGIFL	57.14%	HPV68
14	41-54	ADKILQWSGLGIFL	50.00%	HPV51
15	41-54	ADKILQWSGLGIFL	50.00%	HPV69
16	41-54	ADKILQWSGLGIFL	50.00%	HPV82

Residues that are different from their corresponding residue in the reference sequence are highlighted in red color. Identity indicates the number (%) of residues in the homologous sequences that are identical to the corresponding residue in the reference sequence

Table S4. Validation of 3D structures of the designed SGD58 obtained by the I-TASSER and Robetta and its refinement by the Galaxy Refine (named as I-T Gal) and 3Drefine (named as I-T 3DR)

Model	ProSA	ERRAT	RAMPAGE		
	z-score	Overall quality factor	Favored region	Allowed region	Outlier region
I-TASSER	-5.76	83.2258	249 (78.8%)	44 (13.9%)	23 (7.3%)
I-T Gal1	-5.54	75.6494	282 (89.2%)	23 (7.3%)	11 (3.5%)
I-T Gal2	-5.55	75.1613	281 (88.9%)	22 (7.0%)	13 (4.1%)
I-T Gal3	-5.77	88.889	280 (88.6%)	24 (7.6%)	12 (3.8%)
I-T Gal4	-5.63	79.8701	279 (88.3%)	24 (7.6%)	13 (4.1%)
I-T Gal5	-5.75	77.7419	280 (88.6%)	24 (7.6%)	12 (3.8%)
I-T 3DR1	-5.72	86.8056	261 (82.6%)	35 (11.1%)	20 (6.3%)
I-T 3DR2	-5.72	88.8114	259 (82.0%)	32 (10.1%)	25 (7.9%)
I-T 3DR3	-5.87	88.8112	258 (81.6%)	35 (11.1%)	23 (7.3%)
I-T 3DR4	-5.86	88.8112	259 (82.0%)	30 (9.5%)	27 (8.5%)
I-T 3DR5	-5.89	80.9677	259 (82.0%)	30 (9.5%)	27 (8.5%)
Robetta-Model-1	-5.91	93.2258	306 (96.8%)	10 (3.2%)	0 (0.0%)
Robetta-Model-2	-6.01	96.1291	308 (97.5%)	6 (1.9%)	2 (0.6%)
**Robetta-Model-3**	**-6.65**	**99.0033**	**306 (96.8%)**	**9 (2.8%)**	**1 (0.3%)**
Robetta-Model-4	-5.21	92.1233	307 (97.2%)	6 (1.9%)	3 (0.9%)
Robetta-Model-5	-5.64	98.3871	311 (98.4%)	4 (1.3%)	1 (0.3%)
Robetta M3 Gal1	-6.51	95.9732	311 (98.4%)	4 (1.3%)	1 (0.3%)
Robetta M3 Gal2	-6.29	95.2218	310 (98.1%)	5 (1.6%)	1 (0.3%)
Robetta M3 Gal3	-6.32	97.9798	311 (98.4%)	4 (1.3%)	1 (0.3%)

Robetta M3 Gal4	-6.48	94.6488	310 (98.1%)	5 (1.6%)	1 (0.3%)
Robetta M3 Gal5	-6.57	96.5871	311 (98.4%)	4 (1.3%)	1 (0.3%)
Robetta M3 3DR1	-6.46	97.0968	304 (96.2%)	10 (3.2%)	2 (0.6%)
Robetta M3 3DR2	-6.43	96.7742	305 (96.5%)	9 (2.8%)	2 (0.6%)
Robetta M3 3DR3	-6.37	96.7742	304 (96.2%)	9 (2.8%)	3 (0.9%)
Robetta M3 3DR4	-6.38	96.7742	304 (96.2%)	9 (2.8%)	3 (0.9%)
Robetta M3 3DR5	-6.35	97.0968	304 (96.2%)	9 (2.8%)	3 (0.9%)

The Robetta-Model-3 structure was chosen as the most appropriate model, which is shown in bold; M3 –model 3

Table S5. Validation of 3D structures of the TLR5 obtained by the I-TASSER and Robetta and its refinement by the GalaxyRefine (named as I-T Gal) and 3Drefine (named as I-T 3DR)

Model	ProSA	ERRAT	RAMPAGE		
	Z-score	Overall quality factor	Favored region	Allowed region	Outlier region
I-TASSER	-5.93	79.7619	635 (74.2%)	169 (19.7%)	52 (6.1%)
I-T Gal1	-6.52	68.9781	779 (91.0%)	71 (8.3%)	6 (0.7%)
I-T Gal2	-6.35	73.7864	778 (90.9%)	70 (8.2%)	8 (0.9%)
I-T Gal3	-6.64	73.3414	783 (91.5%)	65 (7.6%)	8 (0.9%)
I-T Gal4	-6.65	70.3163	785 (91.7%)	63 (7.4%)	8 (0.9%)
I-T Gal5	-6.61	72.6176	782 (91.4%)	68 (7.9%)	6 (0.7%)
I-T 3DR1	-6.47	85.967	699 (81.7%)	118 (13.8%)	39 (4.6%)
I-T 3DR2	-6.52	86.3208	708 (82.7%)	109 (12.7%)	39 (4.6%)
I-T 3DR3	-6.53	86.6745	714 (83.4%)	102 (11.9%)	40 (4.7%)
I-T 3DR4	-6.63	86.4387	713 (83.3%)	102 11.9%)	41 (4.8%)
I-T 3DR5	-6.77	87.6179	712 (83.2%)	103 (12.0%)	41 (4.8%)
Robetta-Model-1	-7.2	87.6847	792 (94.7%)	40 (4.8%)	4 (0.5%)
Robetta-Model-2	-7.02	92.0147	781 (93.4%)	48 (5.7%)	7 (0.8%)
Robetta-Model-3	-7.51	89.7277	792 (94.7%)	36 (4.3%)	8 (1.0%)
Robetta-Model-4	-6.91	89.9272	799 (95.6%)	30 (3.6%)	7 (0.8%)
**Robetta-Model-5**	**-6.71**	**92.1472**	**785 (93.9%)**	**45 (5.4%)**	**6 (0.7%)**
Robetta M5 Gal1	-7.11	82.4691	803 (96.1%)	31 (3.7%)	2 (0.2%)
Robetta M5 Gal2	-7.08	85.7673	807 (96.5%)	24 (2.9%)	5 (0.6%)

Robetta M5 Gal3	-7.12	85.5911	803 (96.1%)	28 (3.3%)	5 (0.6%)
Robetta M5 Gal4	-7.14	84.8708	806 (96.4%)	25 (3.0%)	5 (0.6%)
Robetta M5 Gal5	-7.01	85.0307	809 (96.8%)	24 (2.9%)	3 (0.4%)
Robetta M5 3DR1	-6.85	90.6781	764 (91.4%)	63 (7.5%)	9 (1.1%)
Robetta M5 3DR2	-6.92	89.7219	765 (91.5%)	62 (7.4%)	9 (1.1%)
Robetta M5 3DR3	-6.92	89.3720	763 (91.3%)	64 (7.7%)	9 (1.1%)
Robetta M5 3DR4	-6.92	90.0966	762 (91.1%)	64 (7.7%)	10 (1.2%)
Robetta M5 3DR5	-7.02	89.8795	763 (91.3%)	63 (7.5%)	10 (1.2%)

Robetta-Model-5 structure was chosen as the most appropriate model, which is shown in bold.

Table S6. Dis-continuous B-cell epitopes identified in the refined 3D structure of designed vaccine constructs of HPV58 by using Discotope 2.0

S.No	Residue number	Amino acid	Contact number	Propensity score	DiscoTope score
1	12	ASN	5	-3.272	-3.471
2	25	ILE	7	-3.159	-3.601
3	37	ALA	0	-3.037	-2.688
4	38	LYS	5	-2.621	-2.895
5	41	ALA	0	-3.549	-3.141
6	42	ALA	3	-3.414	-3.366
7	55	LYS	6	-3.291	-3.602
8	99	THR	0	-1.665	-1.474
9	101	SER	3	-1.96	-2.079
10	103	SER	0	-2.842	-2.515
11	107	SER	6	-2.739	-3.114
12	130	GLY	5	-2.944	-3.181
13	265	GLY	8	-2.67	-3.283
14	266	ASN	5	-0.617	-1.121
15	269	THR	6	-2.481	-2.886
16	270	ASN	7	-2.764	-3.251
17	284	ALA	1	-3.567	-3.272
18	288	SER	5	-3.336	-3.528

Table S7.

Statistical analysis of the TLR5-SGD58 docking result obtained by HADDOCK

No. of Clusters	HADDOCK score	Total interaction energy (Kcal mol^{-1})	Van der waal energy (Kcal mol^{-1})	Electrostatic energy (Kcal mol^{-1})	Desolvation energy (Kcal mol^{-1})	Restraints violation energy (Kcal mol^{-1})	Buried surface area A 2)
Cluster 1	-29.9 ± 6.0	-34.8 ± 1.2	-46.4 ± 5.5	-513.0 ± 146.5	81.7 ± 32.4	912.9 ± 94.90	1918.1 ± 157.4
Cluster 2	-37.3 ± 8.8	-34.4 ± 0.8	-52.3 ± 6.7	-436.8 ± 100	78.6 ± 8.0	983.3 ± 22.17	1847.4 ± 117.3
Cluster 3	-39.2 ± 4.9	-20.4 ± 0.3	-42.4 ± 8.9	-432.1 ± 12.3	73.1 ± 8.6	949.3 ± 68.33	1720.4 ± 165.1
Cluster 4	-62.5 ± 7.6	-30.0 ± 0.4	-58.3 ± 5.4	-207.8 ± 37.3	43.1 ± 7.9	1192.9 ± 96.93	1914.4 ± 124.4
Cluster 5	-48.1 ± 3.6	-37.1 ± 0.6	-46.0 ± 6.8	-400.7 ± 54.1	74.0 ± 9.7	1003.1 ± 92.31	1732.7 ± 132.7
Cluster 6	-54.2 ± 16.4	-38.5 ± 0.1	-40.2 ± 2.5	-271.7 ± 28.9	56.9 ± 7.5	919.1 ± 128.25	1451.9 ± 71.5
Cluster 7	-61.9 ± 23.9	-21.0 ± 0.4	-55.2 ± 6.8	-179.0 ± 28.3	49.9 ± 11.9	1026.7 ± 45.95	1902.6 ± 247.8
Cluster 8	-60.8 ± 9.5	-36.4 ± 0.3	-46.7 ± 9.5	-270.4 ± 45.0	60.7 ± 2.8	1008.6 ± 167.01	1759.4 ± 121.9
Cluster 9	-60.6 ± 14.6	-30.0 ± 0.3	-59.0 ± 4.8	-196.2 ± 19.9	49.0 ± 11.8	1098.3 ± 114.91	1844.5 ± 122.5
Cluster 10	-42.4 ± 19.9	-3.4 ± 2.0	-68.1 ± 6.8	-254.8 ± 67.5	51.7 ± 15.6	1097.6 ± 117.31	2357.2 ± 101.3
